# The successes and challenges of harmonising juvenile idiopathic arthritis (JIA) datasets to create a large-scale JIA data resource

**DOI:** 10.1186/s12969-023-00839-2

**Published:** 2023-07-13

**Authors:** Saskia Lawson-Tovey, Samantha Louise Smith, Nophar Geifman, Stephanie Shoop-Worrall, Sandra Ng, Michael R. Barnes, Lucy R. Wedderburn, Kimme L. Hyrich, CLUSTER consortium, Melissa Kartawinata, Zoe Wanstall, Bethany R. Jebson, Alyssia McNeece, Elizabeth Ralph, Vasiliki Alexiou, Fatjon Dekaj, Aline Kimonyo, Fatema Merali, Emma Sumner, Emily Robinson, Freya L. Feilding, Andrew Dick, Michael W. Beresford, Emil Carlsson, Joanna Fairlie, Jenna F. Gritzfeld, Athimalaipet Ramanan, Teresa Duerr, Stephen Eyre, Soumya Raychaudhuri, Andrew Morris, Annie Yarwood, Samantha Smith, John Bowes, Paul Martin, Melissa Tordoff, Michael Stadler, Wendy Thomson, Damian Tarasek, Chris Wallace, Wei-Yu Lin, Sarah Clarke, Toby Kent, Thierry Sornasse, Daniela Dastros-Pitei, Sumanta Mukherjee, Jacqui Roberts, Rami Kallala, Helen Neale, John Ioannou, Hussein Al-Mossawi

**Affiliations:** 1 Centre for Genetics and Genomics Versus Arthritis, The University of Manchester, Manchester, UK; 2 National Institute of Health Research Manchester Biomedical Research Centre, Manchester University NHS Foundation Trust, Manchester Academic Health Science Centre, Manchester, UK; 3 School of Health Sciences, Faculty of Health and Medical Sciences, The University of Surrey, Guildford, UK; 4 Centre for Epidemiology Versus Arthritis, The University of Manchester, Manchester, UK; 5 Centre for Health Informatics, The University of Manchester, Manchester, UK; 6 Centre for Translational Bioinformatics, William Harvey Research Institute, Bart’s and the London School of Medicine and Dentistry, Queen Mary University London, London, UK; 7 Centre for Adolescent Rheumatology Versus Arthritis at UCL, University College London Hospitals and Great Ormond Street Hospital, London, UK; 8 Infection, Inflammation and Rheumatology, UCL Great Ormond Street Institute of Child Health, London, UK

**Keywords:** Data harmonisation, Children and young people, JIA

## Abstract

**Background:**

CLUSTER is a UK consortium focussed on precision medicine research in JIA/JIA-Uveitis. As part of this programme, a large-scale JIA data resource was created by harmonizing and pooling existing real-world studies. Here we present challenges and progress towards creation of this unique large JIA dataset.

**Methods:**

Four real-world studies contributed data; two clinical datasets of JIA patients starting first-line methotrexate (MTX) or tumour necrosis factor inhibitors (TNFi) were created. Variables were selected based on a previously developed core dataset, and encrypted NHS numbers were used to identify children contributing similar data across multiple studies.

**Results:**

Of 7013 records (from 5435 individuals), 2882 (1304 individuals) represented the same child across studies. The final datasets contain 2899 (MTX) and 2401 (TNFi) unique patients; 1018 are in both datasets. Missingness ranged from 10 to 60% and was not improved through harmonisation.

**Conclusions:**

Combining data across studies has achieved dataset sizes rarely seen in JIA, invaluable to progressing research. Losing variable specificity and missingness, and their impact on future analyses requires further consideration.

**Supplementary Information:**

The online version contains supplementary material available at 10.1186/s12969-023-00839-2.

## Background

Juvenile idiopathic arthritis (JIA) and uveitis can cause disability and increased comorbidity risk into adulthood if diagnosed late or treated ineffectively [1]. Patients’ day-to-day experiences are varied and cannot be fully captured in clinical trial settings; while real-world data can help answer many research questions, substantial resources and time are needed to collect these. New datasets derived from existing data have many benefits: maximising the availability of larger sample sizes that may not be feasibly collected individually, improving the generalisability and validity of research, and providing multi-disciplinary and multi-centre collaborative opportunities [2].

CLUSTER [3], a UK Research and Innovation Medical Research Council/Versus Arthritis funded multidisciplinary consortium, aims to improve personalised treatments and predict disease outcomes for JIA and JIA-uveitis through bringing together knowledge, studies, and data. It builds on the work of the MRC-funded CHART consortium (Childhood Arthritis Response to Treatment), which explored how to bring together clinical and biological data from 4 UK observational JIA research cohort studies to create a larger unified dataset for analysis of predictors of treatment response. CLUSTER aims to create a large-scale JIA data resource by harmonising existing data collected in clinical trials and real-world JIA cohort studies. Maximising CLUSTER’s clinical and biological data by successfully harmonising multiple datasets is integral to producing robust analyses with maximal power in this rare disease, and facilitates the goals of defining distinct strata across disease and treatment sub-groups.

As heterogeneous datasets are often collected autonomously, for specific analytical objectives and not in coordination, as well as being nuanced (requiring prior knowledge of data capture methods and coding), a key challenge is in managing and combining disparate, non-standardised datasets. Different systems, data structures, and cultural barriers, such as apprehension to share data and restrictions related to ethical, legal and consent-procedures, are also very common [2, 4].

There are many ways to bring together disparate datasets for analysis, such as data pooling or federated data analyses with subsequent meta-analyses of results: all approaches requiring the critical step of data harmonisation. Local data laws and study specific governance may dictate to what extent data pooling and linkage can occur; but where pooling is planned, knowledge of the potential of duplicated subjects across datasets is also key. Data linkage, where data are combined from two or more sources of data with the objective of consolidating facts concerning an individual or event that are not available in any separate record [5], will also enhance the final dataset, although substantial data cleaning, wrangling, and computational resources may be required.

### Objective

In 2021, data from 4 JIA datasets under the CLUSTER umbrella were successfully pooled and made available for analyses. Here, we describe and evaluate the current data harmonisation processes derived as part of CHART and CLUSTER, and highlight how this enables the research and data sharing goals.

## Methods

### Community

CLUSTER is a multi-disciplinary consortium made up of clinicians and researchers in JIA, uveitis, molecular science, epidemiology, bioinformatics, and data science. Through collaborating with the CLUSTER Champions (our patient and public involvement group), our independent international Scientific Advisory Board, and our industry partners, we involve and are able to capture the needs of all relevant stakeholders in our work to improve treatment outcomes for children and young people with JIA and/or JIA uveitis.

### Source data

CHART was the starting point for this ambitious initiative, which required the identification and extraction of data related to JIA treatment and treatment response. Study-specific metadata, including inclusion criteria and data captured in four existing national JIA studies (Table 1; Childhood Arthritis Prospective Study (CAPS) [6], Childhood Arthritis Response to Medication Study (CHARMS) [7], Biologics for Children with Rheumatic Diseases Study (BCRD), BSPAR-Etanercept registry (BSPAR-Et) [8]) was reviewed and brought together into a single, pooled common data model (CDM). These studies were chosen as it was known that they all captured details of JIA disease characteristics, treatment exposures and treatment response, as well as biologic data. These 4 key studies are summarised in Table 1. CLUSTER expands beyond the four CHART studies and also aims to bring in data from other UK studies including JIA-Pathogenesis Study, UK JIA Genetics Consortium (UKJIAGC)) and two clinical trials of new treatments for JIA-Uveitis (SYCAMORE [9] & APTITUDE [10]).

**Table 1:** Summary of key CLUSTER studies

	**BCRD**	**BSPAR-Et**	**CAPS**	**CHARMS**
Study type	Prospective observational	Prospective observational	Prospective longitudinal	Prospective/retrospective observational
Aim of study	To capture information on use, effectiveness, and safety of biologic DMARDs other than etanercept	To evaluate the effectiveness and safety of etanercept treatment in JIA	To identify environmental, clinical and genetic predictors of short- and long-term outcomes in JIA	To understand why some children with JIA respond well to treatments and others do not
Study dates	2010—ongoing	2004—ongoing	2001—ongoing	2006—ongoing
Funders	Versus Arthritis	British Society of Rheumatology, Pfizer	Versus Arthritis	Sport Aiding Medical Research for Kids (SPARKS); MRC, Great Ormond Street Children’s Charity (GOSHCC) and NIHR-GOSH Biomedical Research Centre
Recruiting locations	UK (43 sites)	UK (41 sites)	UK (7 sites)	England (7 sites), Utrecht, Prague
Inclusion criteria	- fulfil ILAR classification criteria for JIA - starting either a non-etanercept biologic drug or MTX or have started this therapy within the past 6 months - no past exposure to biologic DMARDs (MTX comparison arm) - upper age limit of 17	- fulfil ILAR classification criteria for JIA - starting either etanercept or MTX or have started this therapy within the past 6 months - no past exposure to biologic DMARDs (MTX comparison arm)	- aged 16 or below - diagnosed for the first time with inflammatory arthritis affecting one or more joints that have been persistent for more than 2 weeks	- fulfil ILAR classification criteria for JIA - about to start either MTX or TNFi (prospective arm) or already receiving MTX/TNFi for at least 6 months (retrospective arm)
N of participants	~ 1500	~ 2000	~ 1800	~ 1700
Baseline data collection	At the start of MTX or non-etanercept biologic treatment	At the start of MTX/etanercept treatment	At the point of JIA diagnosis	At the start of MTX/TNFi treatment
Baseline data items	- demographics - clinical data - current and previous JIA medication	- demographics - clinical data - current and previous JIA medication	- demographics - family history of arthritis/autoimmune conditions - socio-economic information - clinical data - results of imaging studies - medication history	- demographics - clinical data - laboratory - medication history
Follow-up data collection	6 and 12 months following registration, then annually until at least year 5	6 and 12 months following registration, then annually until at least year 5	6 and 12 months from the first visit to paediatric rheumatology, then annually to year 5, then year 7 and 10	3 (prospective arm only) and 6 months (range 3–12 months) from start of MTX/TNFi
Follow-up data items	- demographics - clinical data - current and previous JIA medication - drug start/stop dates - discontinuation reasons - adverse events	- demographics - clinical data - current and previous JIA medication - drug start/stop dates - discontinuation reasons - adverse events	- demographics - clinical data - results of imaging studies - medication history - medication start/stop dates - reasons for discontinuation	- demographics - clinical data - laboratory - medication history - drug start/stop dates
Blood samples	Yes (untimed—added to study in 2010)	Yes (untimed—added to study in 2010)	Yes (untimed—at any point after enrolment if blood is being taken as part of routine clinical care)	Yes (Baseline, 6 months (range 3–12 months) for prospective arm; any timepoint for retrospective arm)
Sample types collected	DNA, plasma, saliva	DNA, plasma, saliva	Plasma, RNA, DNA, synovial fluid	DNA, PBMC, serum
Percentage of participants who have ever given a sample	59%	40%	69%	76%

*JIA* Juvenile idiopathic arthritis, *DMARDs* Disease modifying anti-rheumatic drugs, *MTX* Methotrexate, *UK* United Kingdom, *ILAR* International League of Associations for Rheumatology, *TNFi* Tumour necrosis factor inhibitors;

### Data harmonisation

#### Establishing the Common Data Model

Key data items to include in the CDM were initially determined through a combination of a literature review, biological plausibility, data and metadata items and by reviewing individual data items and their harmonisation potential. It was deemed important to review the data available on the agreed JIA Core Outcome Variables (COV) [11] in order to calculate established JIA disease activity measures (such as the Juvenile Arthritis Disease Activity Score (JADAS) [12] and the American College of Rheumatology (ACR) Paediatric response criteria [11]). A core and feasible treatment CDM, including common coding, based on clinical measures was defined and agreed.

#### Agreeing the datasets’ purpose

The initial 4 JIA datasets had permissions for data sharing and therefore pooled datasets would be created to facilitate analyses. It was decided that initial distinct datasets of children starting methotrexate (MTX) and tumour necrosis factor inhibitors (TNFi), the 2 most common systemic treatments for JIA, would be created. Each dataset was mapped and transformed to the CDM prior to pooling. Differing levels of granularity were accounted for by combining data using the most inclusive definition. Details on CDM items available in each study are detailed in Table 2, and Supplementary Fig. 1 gives an overview of the data flow and clinical dataset creation process in CLUSTER.

**Table 2:** Availability of CDM elements across the CLUSTER consortium studies

Individual data items	BCRD	BSPAR-Et	CAPS	CHARMS
**Demographics**				
NHS number	Y	Y	Y	Y
Date of birth	Y	Y	Y	Y
Gender	Y	Y	Y	Y
Ethnicity	Y	Y	Y	Y
Height	Y	Y	Y	Y
Weight	Y	Y	Y	Y
**JIA**				
ILAR subtype	Y	Y	Y	Y
Date of JIA diagnosis	Y	Y	N	N
Date of symptom onset	N	N	Y	Y
**COVs**				
AJC	Y	Y	Y	Y
LJC	Y	Y	Y	Y
CHAQ	Y	Y	Y	Y
ESR	Y	Y	Y	Y
CRP	Y	Y	Y	Y
PGA	Y	Y	Y	Y
PGE	Y	Y	Y	Y
Pain VAS	Y	Y	Y	N
**Serology**				
ANA	Y	Y	Y	Y
RF	Y	Y	Y	Y
HLA B27	Y	Y	Y	Y
**MTX/TNFi**				
Start date	Y	Y	Y	Y
Route	N	N	Y	Y
Dose	Y	Y	Y	Y
Stop date	Y	Y	Y	Y
Stop reason	Y	Y	Y	N

*CDM* Common data model, *NHS* National Health Service, *JIA* Juvenile idiopathic arthritis, *ILAR* International League of Associations for Rheumatology, *AJC* Active joint count, *LJC* Limited joint count, *CHAQ* Childhood health assessment questionnaire, *ESR* Erythrocyte sedimentation rate, *CRP* c-reactive protein, *PGA* Physician global assessment, *PGE* Patient/parent global assessment, *VAS* Visual analogue scale, *ANA* Anti-nuclear antibody, *RF* Rheumatoid factor, *HLA B*27 Human leukocyte antigen B27, *MTX* Methotrexate, *TNFi* Tumour necrosis factor inhibitors

Our source datasets were all longitudinal and facilitated time point analyses for the study of treatment response. Optimal time points were defined based on individual study designs and the collection time points of data items; baseline (drug start) and 6 months were selected as the best fit to the clinical research question and the data available. As each study allowed flexibility around data collection to fit around routine hospital visits, a window of 3 months before drug start for baseline and 3–12 months for follow-up were defined to capture data that was collected around the chosen time points; if there were multiple visits in this 3–12 month period, the visit closest to 6 months was selected. Some studies collected data more frequently and if a patient had multiple entries of data within the time point window, their closest measurement to baseline/6 months was used.

#### Data harmonisation hurdles

Some key variables of interest were not available in all studies (e.g. pain visual analog scale (VAS), antinuclear antibodies (ANA) and human leukocyte antigen (HLA) B27), either at the study level or missing at the individual level. Where this occurred, data was recorded as missing and plans to use either complete case analyses or various imputation methods (where appropriate), such as multiple imputation by chained equations (MICE), would be used. Each drug-specific dataset had the same patient inclusion/exclusion criteria applied (diagnosis of JIA, classified by International League Against Rheumatism (ILAR) subtype; treatment-naïve to MTX/TNFi (dataset dependent); MTX/TNFi continued for at least 3 months after starting; at least one COV with no missing data). These were as broad as possible to give flexibility in deciding dataset specificity based on analysis requirements (e.g. reducing time point parameters for timepoint 2, i.e. selecting COV data from 4–8 months after treatment start instead of 3–12 months).

#### Identifying duplicate participants

Given that there was significant overlap in the time period for data collection and location of study sites across the 4 studies, there was a high possibility of the same participants taking part in more than one study. Conducting data linkage in a well-organised secure environment is essential, and using unique identifiers as linkage criteria (deterministic linkage [13]) can reduce the probability of incorrectly linking individuals. However, limitations such as identifier accuracy can result in missed matches and unseen bias [14].

An approach to identify these subjects, and a strategy for combining individual-level data, was needed. All studies captured either the UK National Health Service (NHS) (England, Wales, Northern Ireland) or Community Health Index (CHI) number (Scotland), a number unique to each person in the UK, assigned at birth or at point of immigration/registration with the NHS. The NHS/CHI number is a unique identifier which falls under protection from UK General Data Protection Regulation (GDPR) and therefore can facilitate data transfer and the identification of duplicates but could not be shared with third parties.

We used OpenPseudonymiser [15], an open source software, hosted in secure research data storage settings at the University of Manchester and University College London which are both certified to NHS Data Security & Protection Toolkit standards. OpenPseudonymiser processes CSV files and pseudonymises identifiable fields such as NHS numbers. It checks the validity of the NHS number and encrypts it to produce a string of output characters, known as the digest. A salt file means the digest output is unique to CLUSTER – any project also using OpenPseudonymiser on the same list of NHS numbers will not produce the same pseudonymised output, unless they use our salt file. The digest can then be used to link data across our cohort studies, as the same NHS number with the same encryption will produce the same digest.

#### Dealing with duplicate participants

Each participant was assigned a unique CLUSTER identifier based on their digest; this digest is unique for each individual and identifies which children participated in multiple studies. These matches were also confirmed additionally against existing known duplicate lists (created from internal NHS number and/or genetic comparisons), where any identified errors in NHS numbers, sample labels, and duplicate pairs were corrected.

Not all duplicate records resulted in duplicate data, as children could enter the various studies at different stages of their disease (e.g. at disease onset to CAPS, at start of etanercept to BSPAR-ETN). Where duplicate records of the same child containing common data across the same treatment were identified, we agreed on a broad set of hierarchical rules to decide which records to keep (e.g. if a child had records in CHARMS and CAPS, we kept their CHARMS record as CHARMS was set up to evaluate treatment response and overall the data had lower missingness across the core variables), as shown in Supplementary Fig. 2. We considered merging data cross-study if an individual had duplicate records with missing data points; this could have flaws which impact on analysis, such as treating data from different dates/studies during the treatment pathway as though from the same time point/study, and would involve making individual-level decisions on which data to keep for hundreds of individuals. We decided to take a pragmatic approach and keep/remove duplicate data on an individual-level, not a variable-level.

#### Data platform

CLUSTER facilitates data access to consortium members and external researchers using the open source tranSMART data warehouse platform [16] (Supplementary Fig. 3). TranSMART presents clinical and biological data in an integrated and easily accessible web interface which facilitates data exploration, cohort identification, and complex queries for hypothesis generation and validation. The final CLUSTER treatment datasets will be stored in tranSMART and a data access policy is in place.

## Results

### Data harmonisation

**Fig. 1:**
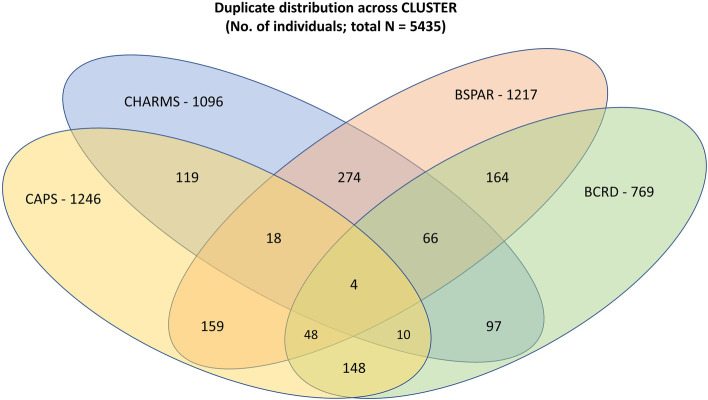
Distribution of individuals with more than one record at any timepoint (“duplicates”) across CLUSTER studies

Overall, a total of 7013 records (from 5435 individuals) were identified across the 4 studies; 2882 records (41%, corresponding to 1304 individuals) represented the same child across the 4 studies: 197 individuals had multiple treatment records within 1 study, 961 across 2 studies, 142 in 3, and 4 children had records in all 4 studies. The crossover of duplicate records is shown in Fig. 1.

Two harmonised, pooled clinical datasets have been created: MTX starters (*n* = 2889) and TNFi starters (*n* = 2401), after removing 250 MTX and 605 TNFi duplicate records respectively; 1018 patients are included in both datasets having started both treatments at different, consecutive timepoints. Both harmonised datasets are accessible on the tranSMART platform with accompanying data and metadata documentation.

Table 3 describes key clinical characteristics in the CLUSTER MTX and TNFi datasets compared to the source cohort studies. Key characteristics are broadly similar across the studies. Where there are more noticeable differences, these can potentially be explained through our hierarchical choice on which data to keep out of duplicate records and the nature of each CLUSTER dataset (i.e. each dataset wants to capture patients who have taken MTX/TNFi for the first time).

**Table 3:** Key clinical characteristics across the CLUSTER harmonised datasets and the original cohort studies

		**CLUSTER MTX dataset**	**CLUSTER TNFi dataset**	**BSPAR-Et/BCRD**^**a**^	**CAPS**^**a**^	**CHARMS**^**a**^
**Total N**		2899	2401	3608	1701	1956
**Gender**	Male	929 (32.1)	776 (32.3)	1138 (31.5)	600 (35.3)	632 (32.3)
	Female	1959 (67.6)	1616 (67.3)	2452 (68.0)	1101 (64.7)	1312 (67.1)
	Missing	11 (0.3)	9 (0.4)	18 (0.5)		12 (0.6)
**Age at JIA onset (mean, SD)**		6.6 (4.3)	6.9 (4.5)	7.0 (4.5)	7.1 (4.4)	6.0 (4.1)
**White ethnicity**	Caucasian	2530 (87.3)	2040 (85.0)	3044 (84.4)	1365 (80.3)	1768 (90.4)
	Non-Caucasian	363 (12.5)	351 (14.6)	527 (14.6)	336 (19.8)	182 (9.3)
	Missing	6 (0.2)	10 (0.4)	37 (1.0)		6 (0.3)
**JIA subtype at baseline**	Systemic JIA	219 (7.6)	132 (5.5)	300 (8.3)	105 (6.2)	141 (7.2)
	Oligoarthritis—persistent	566 (19.5)	296 (12.3)	426 (11.8)	676 (39.7)	333 (17.0)
	Oligoarthritis—extended	434 (15.0)	421 (17.5)	634 (17.6)	90 (5.3)	396 (20.3)
	Polyarthritis—RF negative	911 (31.4)	801 (33.4)	1173 (32.5)	322 (18.9)	657 (33.6)
	Polyarthritis—RF positive	172 (5.9)	191 (8.0)	307 (8.5)	59 (3.5)	107 (5.5)
	Enthesitis-related arthritis	172 (5.9)	235 (9.8)	306 (8.5)	82 (4.8)	116 (5.9)
	Psoriatic JIA	203 (7.0)	156 (6.5)	227 (6.3)	114 (6.7)	107 (5.5)
	Undifferentiated arthritis	138 (4.8)	109 (4.5)	103 (2.9)	173 (10.2)	15 (0.7)
	Missing	84 (2.9)	60 (2.5)	132 (3.7)	80 (4.7)	84 (4.3)
**Active joint count (mean, SD) at baseline**		7.4 (8.2)	2.5 (4.7)	6.0 (7.9)	4.4 (6.9)	7.1 (7.8)
**Limited joint count (mean, SD) at baseline**		5.7 (7.6)	3.2 (6.8)	5.1 (7.4)	3.4 (5.8)	5.7 (7.4)
**Uveitis**	Yes	311 (10.7)	428 (17.8)	559 (15.5)	241 (14.2)	273 (14.0)
	No	2073 (71.5)	1768 (73.6)	2762 (76.5)	1278 (75.1)	1291 (66.0)
	Missing	515 (17.8)	205 (8.6)	287 (8.0)	182 (10.7)	392 (20.0)

All numbers are N(%) unless stated otherwise

*JIA* Juvenile idiopathic arthritis, *RF* Rheumatoid factor, *SD* Standard deviation, *MTX* Methotrexate, *TNFi* TNF inhibitors

^a^the numbers from these studies include all recruited patients, regardless of therapeutic regimen

### Data missingness

**Fig. 2:**
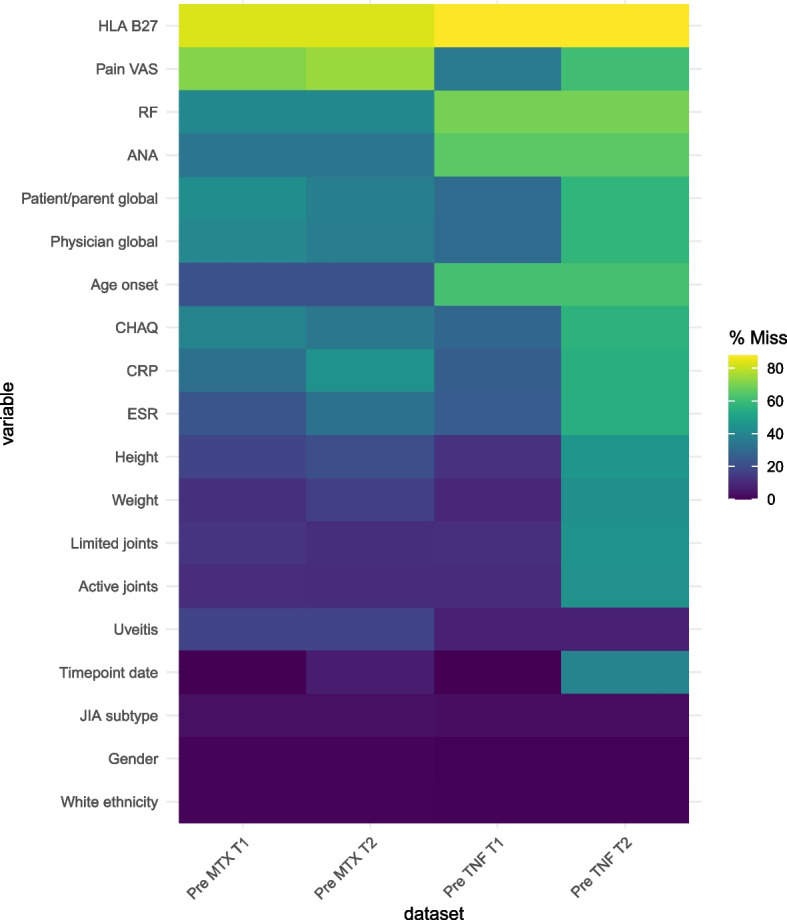
Percentage of missingness across key variables in the CLUSTER MTX and TNF datasets *MTX* Methotrexate, *TNF* Tumour necrosis factor inhibitors, *T1* Timepoint 1 (closest values to baseline; allowed -3 months to drug start), *T2* Timepoint 2 (closest value to 6 months after drug start; allowed 3–12 months after drug start), *JIA* Juvenile idiopathic arthritis, *RF* Rheumatoid factor, *HLA B27* Human leukocyte antigen B27, *ANA* Anti-nuclear antibody, *CHAQ* Childhood health assessment questionnaire, *ESR* Erythrocyte sedimentation rate, *CRP* C-reactive protein, *VAS* Visual analogue scale

Although combining datasets increased the sample size considerably, it did not significantly improve levels of missingness, despite attempting to choose the record with the lowest missingness for children with duplicate records (Fig. 2). This likely reflects the fact that these real-world data were extracted from the same medical records and deposited into parallel studies with a high level of existing overlap in their case report forms. Some missingness was expected, particularly in variables that are not routinely collected in clinical visits, and in many cases, higher levels of missingness related to differences in the individual study design (e.g., ANA and HLA-B27 not captured in all studies).

### Data crossover

A significant number of these participants also have stored biological samples – the percentages of children who gave biological samples in each original study are shown in Table 1. CLUSTER is currently conducting several analyses which include biological data – by using the CLUSTER ID to identify children included in multiple analyses, we can see the extent of data overlaps within CLUSTER. For example, our ongoing genome-wide association study (GWAS) on JIA patients who started first-line MTX includes 44% of patients in the MTX dataset, and 19% of those in the TNFi dataset, and our ongoing uveitis HLA-B27 fine-mapping study includes 48% and 33% of the MTX and TNFi cohorts, respectively.

## Discussion

### Successes

Data from over 5400 individual patients with JIA were harmonised to create prospective detailed JIA treatment datasets at a scale rarely seen – the highest number of participants in one of the contributing cohort studies is around 2000 across both MTX and etanercept (BSPAR-Et), compared to 2899 in this MTX and 2401 in this TNFi dataset. Many of these studies continue to recruit patients and collect further data; by logging the processes to create the existing dataset, it can be updated at intervals to expand it further. This is invaluable to progressing meaningful JIA research, particularly into personalised treatments and disease outcomes. Integration has added depth, enables big-data approaches such as machine learning, and highlights inconsistencies that would not be apparent in the individual datasets.

Encrypting and matching duplicates led to improvements in identifying erroneous NHS numbers and biological sample labels in the original studies. Using encrypted NHS numbers and pseudonymised study IDs maximised data usage through pooling individual treatment data from multiple sources and time points to create a more complete picture of a patient’s treatment pathway. This process can also bring in further data as it is generated or discovered. With a common unique identifier facilitating data pooling, larger datasets can now be anonymised and shared with external collaborators and third parties.

Building CLUSTER into a multi-disciplinary community was key in achieving our goals, particularly the early involvement of informatics and data science professionals. These datasets also provide the opportunity to expand our community and link with established consortia such as IMID-BIO-UK [17] to facilitate cross-disease comparison. The additional inclusion of public datasets from the Gene Expression Omnibus (GEO) repository will allow cross-comparison and confirmation in external datasets.

### Challenges

Whilst the duplicate patient identification process appears to be accurate, mismatches were identified. As some of these resulted from inaccurate recording of the NHS number in the original study database, it is possible to unknowingly miss duplicate pairs. It is also possible to miss duplicate pairs if a patient is missing an NHS number in one study, though this is a rare occurrence.

Harmonising data is a laborious process as each study is nuanced and significant data cleaning is needed to account for this. Losing specificity impacts detail available for analysis and broad duplicate removal rules could be disadvantageous. For example, where duplicate records existed, we kept CHARMS data over other studies, but automatically lost some pain VAS outcome measures as these are not collected in CHARMS, and the records were retained at the person level and not at the variable level. The impact of this may be an area for future data science research as the impact on our prediction studies has not been fully realised. We also lost granularity, e.g. ethnicity had to be coded in the final CLUSTER dataset as Caucasian/Non-Caucasian as that was the least granular classification across all studies, but much more detailed ethnicity information is available in some studies.

When creating harmonised datasets from existing observational studies, missing data are expected. Our aim was to maximise dataset sizes by avoiding limiting to complete cases only; something that would only be needed for some comprehensive measures of JIA disease activity change. Including those with some missing data retains statistical power and reduces potential biases. However, this could mean that established and validated JIA disease scores cannot be used in some circumstances if missing data are high; though this issue would also exist in the source data. If we choose to apply imputation methods, we can use all available data and make unbiased estimates of expected values, thereby providing more validity than ad hoc approaches to missing data while preserving our sample sizes and power. Imputation methods could also facilitate the inclusion of certain variables within larger analyses that were not collected at all in the source data.

## Conclusion

Data pooling and harmonisation are important tools for research, enabling the development of larger, richer datasets which contain detailed treatment response data across patients’ treatment pathways. CLUSTER has succeeded in integrating large, complex JIA datasets and provides a useful reference to similar future projects. Agreeing a framework pre-integration was essential – focusing on a specific, well-defined research question for each dataset meant they were manageable and tailored to their intended use, whilst easily enabling adjustments. Additionally, CLUSTER’s collaborative process was pivotal as data integration on this scale requires a committed, knowledgeable, and diverse community.

However, there are many challenges to consider: time/costs, false linkage, loss of detail, the introduction of errors, systematic biases, and missingness. It is important these limitations are recognised to avoid misinterpretation of findings. Transparent and consistent reporting and appraisal of linked datasets can assist in improving future data collection, coding practices and linkage processes. This again highlights the importance of standardised data collection in the clinical setting.

Ongoing and future studies in JIA should focus on FAIR (findable, accessible, interoperable, reusable) principles [18] to ensure data utility in research outside of initial study plans. One potential solution is to use a consensus-agreed core outcome dataset, which is then widely implemented in clinical care, captured in electronic patient records that are compatible with fast, efficient data download (with appropriate consent for research) such as the one created by CAPTURE-JIA [19].

## Supplementary Information

**Additional file 1.** Supplementary figures. 

## Data Availability

Information regarding access to CLUSTER data can be found on the CLUSTER website (https://www.clusterconsortium.org.uk/researchers/cluster-datasets-and-data-access/). CLUSTER are open to sharing data with other researchers through our secure tranSMART platform. Researchers are welcome to get in touch with CLUSTER to discuss their project and potential application for data access, as well as access to more information about the contents of CLUSTER datasets through documentation such as a data dictionary. The OpenPseudonymiser software and source code are available here: https://www.openpseudonymiser.org/. Researchers are welcome to get in touch with any further questions. The OpenPseudonymiser software and source code are available here: https://www.openpseudonymiser.org/. Researchers are welcome to get in touch with any further questions.

## Abbreviations

JIA: Juvenile idiopathic arthritis
CLUSTER: Childhood arthritis and its associated uveitis: STratification via Endotypes and mechanism to deliveR benefit
UK: United Kingdom
MRC: Medical Research Council
CHART: Childhood Arthritis Response to Treatment
MTX: Methotrexate
TNFi: Tumour necrosis factor inhibitors
NHS: National Health Service
ANA: Anti-nuclear antibodies
HLA-B27: Human leukocyte antigen B27
ID: Identification
GWAS: Genome-wide association study
IMID-BIO-UK: Immune-Mediated Inflammatory Disease Biobanks United Kingdom
GEO: Gene Expression Omnibus
VAS: Visual analog scale
FAIR: Findable, accessible, interoperable, reusable
CAPTURE-JIA: Consensus derived, Accessible (information), Patient-focused, Team-focused, Universally-collected (UK), Relevant to all and containing Essential data items
CAPS: Childhood Arthritis Prospective Study
CHARMS: Childhood Arthritis Response to Medication Study
BCRD: Biologics for Children with Rheumatic Diseases Study
BSPAR-Et: British Society of Paediatric and Adolescent Rheumatology Study Etanercept registry
CDM: Common data model
UKJIAGC: UK JIA Genetics Consortium
SYCAMORE: Randomised controlled trial of the clinical effectiveness, SafetY and Cost effectiveness of Adalimumab in combination with MethOtRExate for the treatment of juvenile idiopathic arthritis associated uveitis
APTITUDE: A phase II trial of Tocilizumab in anti-TNF refractory patients with JIA associated uveitis
COV: Core outcome variable
JADAS: Juvenile Arthritis Disease Activity Score
ACR: American College of Rheumatology
ILAR: International League Against Rheumatism
CHI: Community Health Index
GDPR: General Data Protection Regulation
CSV: Comma separated value

## Declarations

### Disclaimer

The views expressed in this publication are those of the authors and not necessarily those of the National Health Service, the NIHR, or the Department of Health.
