# Supplementary material for: The successes and challenges of harmonising juvenile idiopathic arthritis (JIA) datasets to create a large-scale JIA data resource
Source: Pediatr Rheumatol Online J. 2023 Jul 13;21:70. doi: 10.1186/s12969-023-00839-2 (PMC10339614; doi:10.1186/s12969-023-00839-2)
Supplement: Supplementary file 1 — Additional file 1. Supplementary figures. [file 12969_2023_839_MOESM1_ESM.docx]

**CLUSTER Data Harmonisation paper – Supplementary Figures**

**Supplementary Figure S1: Overview of CLUSTER clinical data flow**


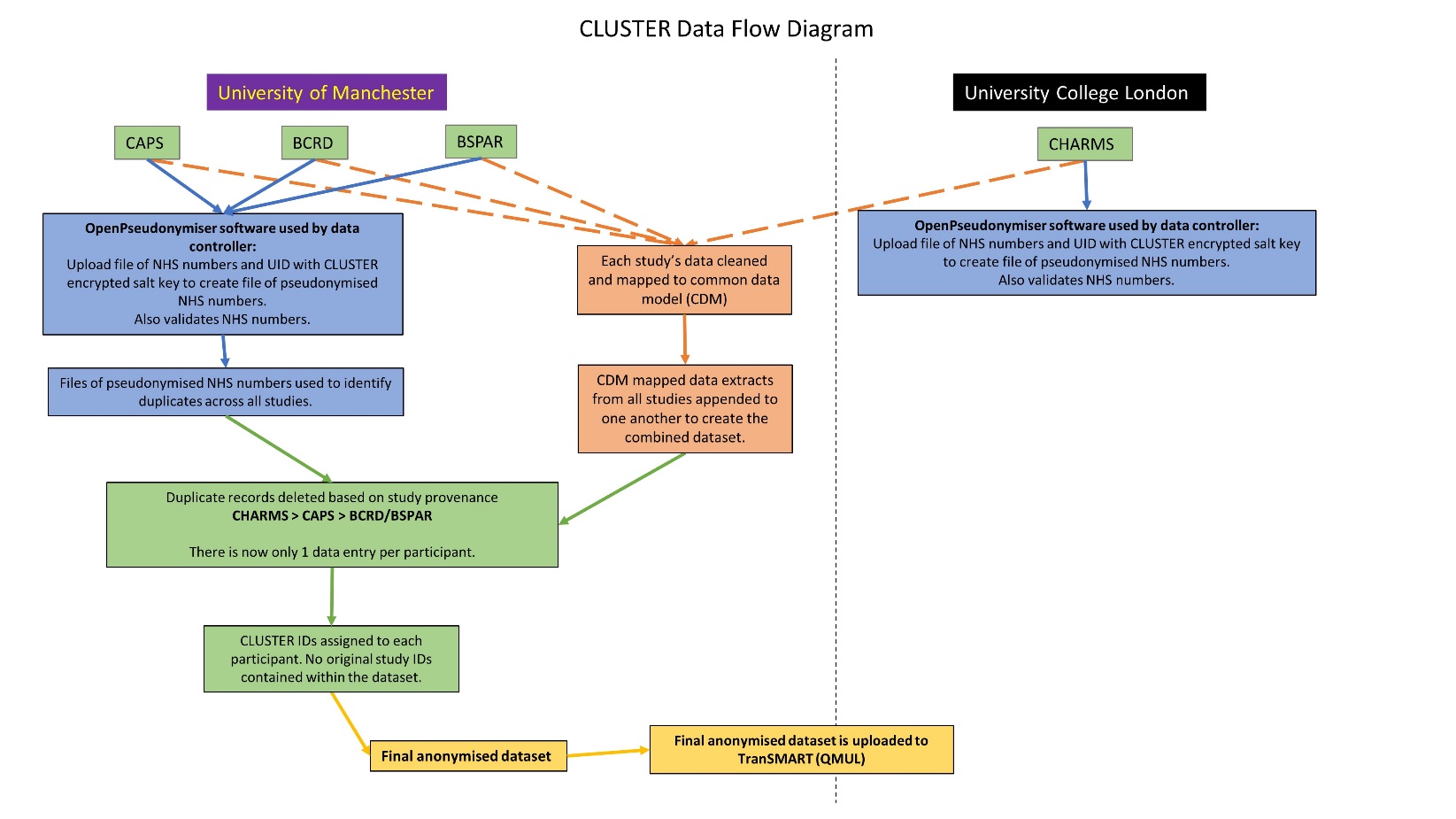


**Supplementary Figure S2: Overview of CLUSTER duplicate identification/removal process**


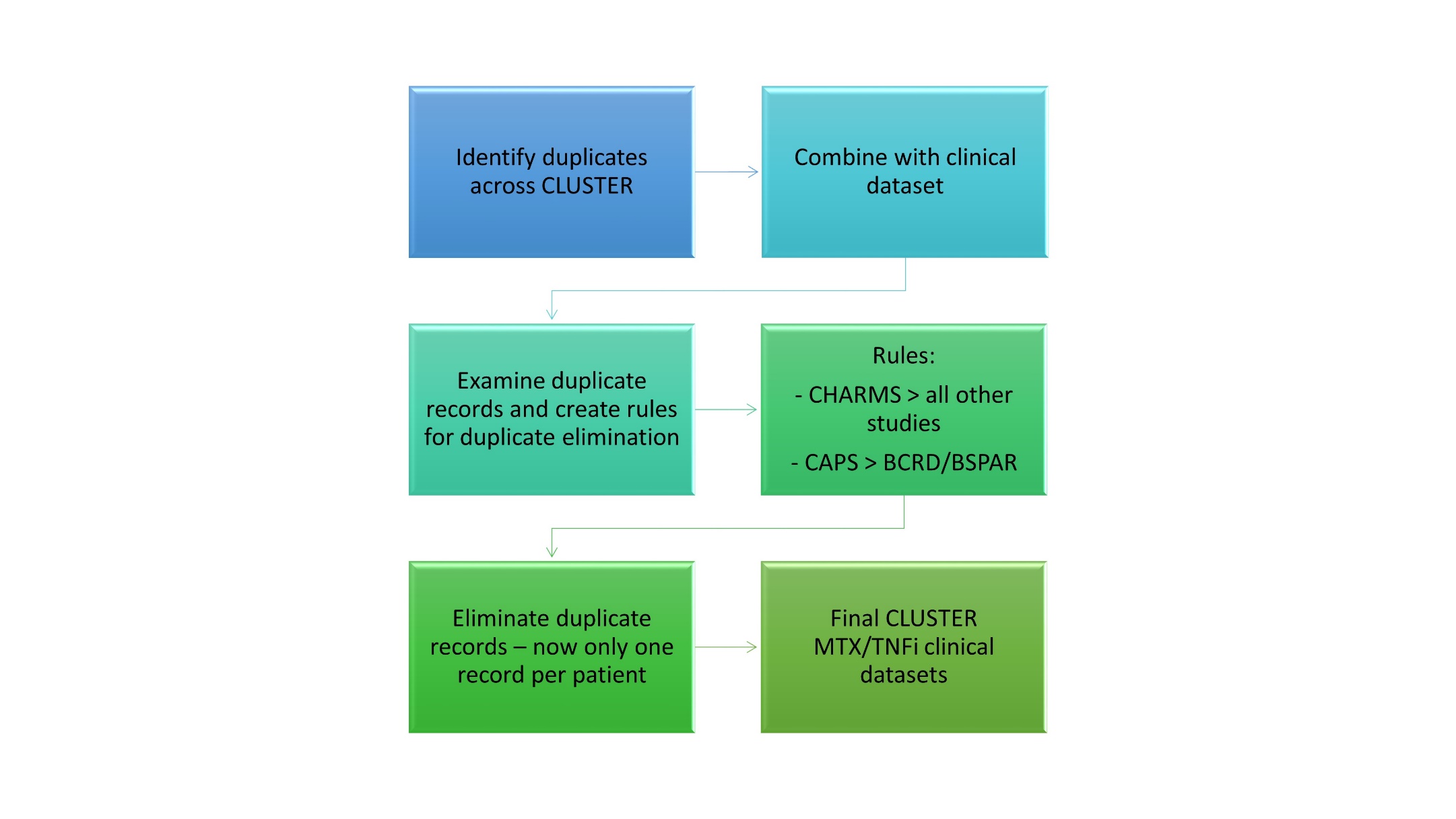


**Supplementary Figure S3: Overview of the CLUSTER tranSMART data infrastructure**

**
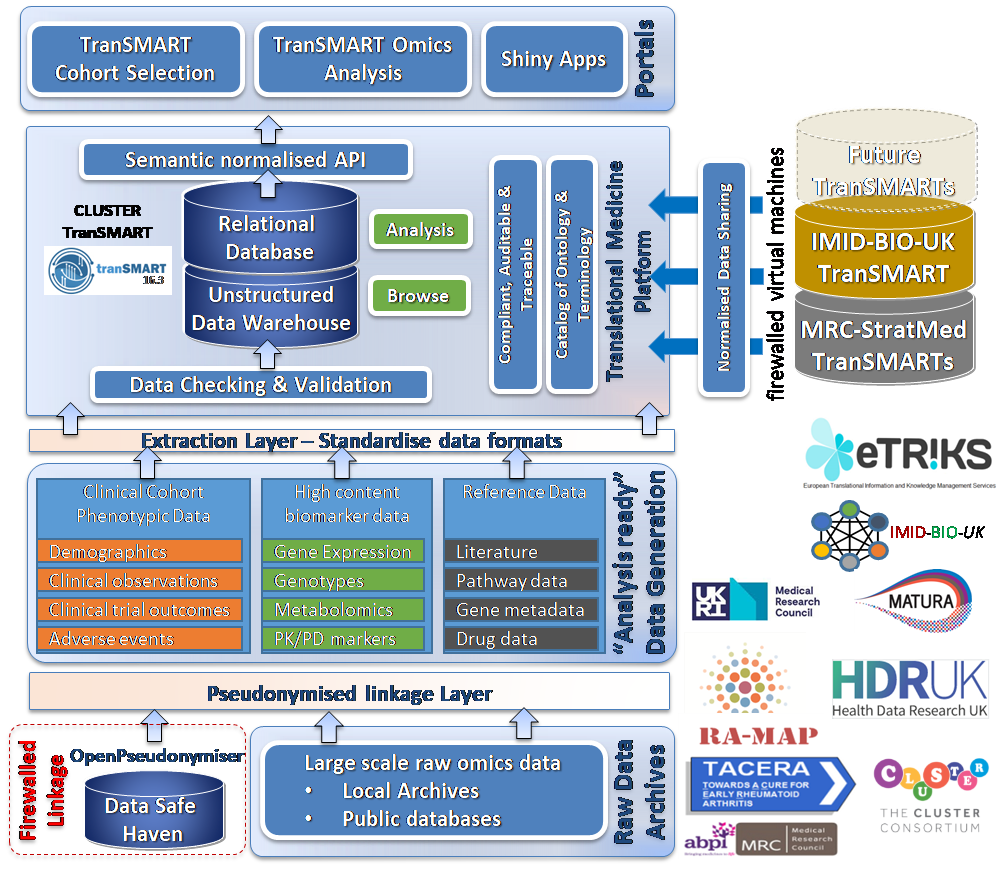
**
